# A non-field analytical method for gas dissolution under forced compression

**DOI:** 10.1038/s41598-022-07430-x

**Published:** 2022-03-01

**Authors:** Vladimir Kulish, Vladimír Horák

**Affiliations:** 1 School of General Engineering, Beihang University, Beijing, China; 2 Department of Mechanical Engineering, Faculty of Military Technology, University of Defence, Brno, Czech Republic

**Keywords:** Mechanical engineering, Thermodynamics

## Abstract

This paper presents an extension of the non-field analytical method—known as the method of Kulish—to model gas dissolution into a liquid due to forced compression. Solutions are obtained for the time evolution of pressure (and, hence, mass concentration) at the gas–liquid interface. These solutions are in the form of series with respect to fractional differ-integral operators. The asymptotic solutions for the two limiting cases of compression—slow and fast compression—have been established as well. Then several particular examples of the law of gas volume variation are considered. Among them, the law of a linear volume variation is the most interesting for practical purposes, in which case numerical values of the dimensionless pressure as a function of dimensionless time are provided.

## Introduction

This paper presents an extension of the non-field analytical method—known as the method of Kulish^1^—to model gas dissolution into a liquid due to forced compression.

The method has been first proposed by Kulish and Lage^2^ more than 2 decades ago and since then was successfully employed for solving various problems in heat and mass transfer^3^, fluid mechanics^4,5^, and other areas^6,7^. In one of the recent works, the method received its full theoretical justification, whereas it has been shown that the solutions it renders can always be represented in the form of either series with respect to fractional differ-integral operators^8^, or compact fractional differ-integral operators with improved convergence^9^.

The problem, considered in this study, belongs to a large class of problems on the dynamics of phase interaction—ranging from modelling the process of boiling^10^, to combustion of solid propellants^12^, and even to applications in military technology (e.g., the non-equilibrium model of depressurisation in two-phase fluids to study the performance of air-guns used in combat training)^11^. In all of these cases, to tackle this type of problems, various methods are used, but all of them render approximate solutions only^10–12^. In contrast, the method, employed in this study, renders exact solutions. Moreover, the mentioned approximate methods are much more laborious in contrast to the easy-to-apply formulae, obtained in this study for the first time. In particular, the inequality for estimating the lower pressure limit, established in “Asymptotic solutions for the two limiting cases of compression” section, may be very useful for practicing engineers.

“Problem formulation” section is fully devoted to the problem formulation and assumptions made. Then, in “Solution procedure” section, solutions are obtained for the time evolution of pressure (and, hence, mass concentration) at the gas–liquid interface. These solutions are in the form of series with respect to fractional differ-integral operators. In “Asymptotic solutions for the two limiting cases of compression” section, the asymptotic solutions for the two limiting cases of compression—slow and fast compression—have been established. In addition, an important inequality for estimating the pressure is as well provided. “Some examples” section presents several particular examples of the law of gas volume variation. Among them, the law of a linear volume variation is the most interesting for practical purposes, in which case numerical values of the dimensionless pressure as a function of dimensionless time are provided.

The solution method considered in this paper, in addition to direct practical interest, seems to be useful from a methodological point of view, since the approach proposed here is applicable to the study of more complex problems of the dynamics of phase interaction.

## Problem formulation

The process of gas dissolution under forced compression, if convective currents are absent, can be modelled by the following set of equations^11^:

1a $$\frac{d}{dt}\left[ {V_{0} f\left( {\frac{t}{\theta }} \right)\frac{PM}{{RT}}} \right] = FD\left. {\frac{\partial C}{{\partial x}}} \right|_{x = 0}$$

1b $$- \sqrt D \frac{\partial C}{{\partial x}}|_{x = 0} = \frac{{d^{1/2} }}{{dt^{1/2} }}\left( {C_{s} - C_{0} } \right),0 < t < \theta$$

1c $$C_{t = 0} = C_{0} ,P_{t = 0} = P_{0} = C_{0} |_{\kappa } ,P = C_{s} |_{\kappa }$$

where ${V}_{0}$ is the initial volume of the gas, $\theta$ is the compression time to zero volume, $f$ denotes a given volume change function [$f(0)=1$; $f(1)=0$], $M$ is the molar mass of the gas, $R$ is the universal gas constant, $D$ is the gas diffusivity in the liquid, $\kappa$ is the Henry coefficient, $F$ is the gas contact area, and $C$ denotes the concentration of the dissolved gas.

In Eq. (1b), the operation of fractional differ-integration is defined in the Riemann–Liouville sense as^13^

2 $$\frac{{d}^{\nu }g(t)}{d{t}^{\nu }}={\mathfrak{D}}^{\nu }g(t)=\frac{1}{\Gamma (1-\nu )}\frac{d}{dt}{\int }_{0}^{t}\frac{g(\zeta )d\zeta }{(t-\zeta {)}^{\nu }},-\infty <\nu <1$$

The *x*-axis is directed downward from the surface and the depth of the liquid is taken to be infinite.

The gas temperature is assumed to remain constant, which suggests a fairly slow compression. If necessary, the non-isothermality of the process can be easily taken into account by making an appropriate change in the function $f(t/\theta )$. Such a kind of process is realised, for example, during thermal expansion of a liquid in a closed vessel containing a gas cavity.

Equation (1a) expresses the change in the mass of the gas volume due to diffusion through the gas–liquid interface. The mass flux is unambiguously related to the change in concentration at the boundary^2^ by means of relation (1b), which makes it unnecessary to consider the process of mass transfer in the region $x>0$. It is required to determine the pressure $P$ in the "cushion" as a function of time.

## Solution procedure

Upon introducing dimensionless variables $p=c=C/{C}_{0}=P/{P}_{0}$, $\xi =x/\sqrt{D\theta }$, $\tau =t/\theta$, $\lambda =\kappa RT\sqrt{D\theta }/(M{V}_{0})$, the set of Eq. (1) becomes

3a $$\frac{d}{d\tau }\left[cf(\tau )\right]=\lambda \frac{\partial c}{\partial \xi }{|}_{\xi =0}$$

3b $$-\frac{\partial c}{\partial \xi }{|}_{\xi =0}=\frac{{d}^{1/2}}{d{\tau }^{1/2}}(c-1),c{|}_{\tau =0}=1$$

Eliminating $(\partial c/\partial \xi {)}_{\xi =0}$ from Eqs. (3a) and (3b) renders the equation for the dimensionless concentration—and, hence, the dimensionless pressure $p(\tau )$—at the interface:

4a $$\frac{d}{d\tau }[f(\tau )p]+\lambda \frac{{d}^{1/2}}{d{\tau }^{1/2}}(p-1)=0$$

4b $$p{|}_{\tau =0}=1$$

Applying operator ${\mathfrak{D}}^{-1}={\int }_{0}^{\tau }(\cdot )d\tau$ to Eq. (4a) and taking into account the initial condition (4b) together with^13^

5 $${\mathfrak{D}}^{\nu }{t}^{\mu }=\frac{\Gamma (\mu +1)}{\Gamma (\mu +1-\nu )}{t}^{\mu -\nu },\mu >-1,\nu <1$$

yields an alternative form of Eq. (4a), which is more useful for practical purposes:

6 $$f(\tau )p+\lambda {\mathfrak{D}}^{-1/2}p=1+2\lambda \sqrt{\frac{\tau }{\pi }}$$

The latter equation is an integral equation with respect to the unknown function $p$. However, here it will be treated as a differential equation of fractional order. This makes it possible to take into account the specifics of the fractional differ-integration in the most complete way in the case when $f(\tau )$ is given by the series

7 $$f(\tau )=\stackrel{\infty }{\sum_{n=0}}{b}_{n}{\tau }^{n/2},{b}_{n}=\text{const},{b}_{0}=1$$

It is necessary to point out here that the latter expression belongs to the series representation of fractional differ-integral operators with improved convergence. Convergence of such a representation has been studied in much detail in a recent work by the authors^9^. It has been shown there that fractional power series of the form given by Eq. (7) provide much better convergence in comparison with the corresponding standard power series expansion (series of integer powers). The latter circumstance is of great importance in case when asymptotic solutions are to be found for large values of *t*.

Now, because the fractional operator ${\mathfrak{D}}^{-1/2}$ transforms a power function into a power function [see Eq. (5)], the solution can be expressed in the series form as

8 $$p\left( \tau \right) = \mathop {\mathop \sum \limits_{n = 0} }\limits^{\infty } a_{n} \tau^{n/2} ,a_{0} = 1,$$

where the coefficients ${a}_{n}$ are found from the recurrent relations

9 $${a}_{1}=-{b}_{1},\stackrel{n+1}{\sum_{k=0}}{a}_{n+1-k}{b}_{k}+\lambda {a}_{n}\frac{\Gamma \left(\frac{n}{2}+1\right)}{\Gamma \left(\frac{n}{2}+\frac{3}{2}\right)}=0$$

## Asymptotic solutions for the two limiting cases of compression

In this section, asymptotic solutions for the two limiting cases of compression are obtained, namely—slow compression ($\lambda \gg 1$) and fast compression ($\lambda \ll 1$).

For $\lambda \gg 1$ (slow compression), the solution of Eq. (6) can be found in the form^9^

10 $$p=\stackrel{\infty }{\sum_{0}}{\lambda }^{-n}{p}_{n}$$

Upon substituting Eq. (10) into (6) and equating the expressions at the equal powers of ${\lambda }^{-n}$, the solution becomes

11 $$p=1+\frac{1}{\lambda }{\mathfrak{D}}^{1/2}[1-f(\tau )]-\frac{1}{{\lambda }^{2}}{\mathfrak{D}}^{1/2}f(\tau ){\mathfrak{D}}^{1/2}[1-f(\tau )]+\cdots$$

As can be seen from the latter equation and Eq. (2), for $\tau \to 1$, the first two terms in the series provide an asymptotic solution for $\lambda \gg 1$.

For $\lambda \ll 1$ (slow compression), an asymptotic solution can be found upon rewriting Eq. (6) in the form

12 $$\left[1+\frac{\lambda }{f(\tau )}{\mathfrak{D}}^{-1/2}\right]p=\frac{1}{f(\tau )}\left(1+2\lambda \sqrt{\frac{\tau }{\pi }}\right)$$

Then the formal solution

13 $$p=\frac{1}{1+\lambda {f}^{-1}(\tau ){\mathfrak{D}}^{-1/2}}\frac{1}{f(\tau )}\left(1+2\lambda \sqrt{\frac{\tau }{\pi }}\right)$$

can be found upon expanding the operator into power series with respect to $\lambda$ (or, which is the same, with respect to ${\mathfrak{D}}^{-1/2}$)^9^:

14 $$p=\left[\frac{1}{f(\tau )}-\lambda \frac{1}{f(\tau )}{\mathfrak{D}}^{-1/2}\frac{1}{f(\tau )}+{\lambda }^{2}\frac{1}{f(\tau )}{\mathfrak{D}}^{-1/2}\frac{1}{f(\tau )}{\mathfrak{D}}^{-1/2}\frac{1}{f(\tau )}-\cdots \right]\left(1+2\lambda \sqrt{\frac{\tau }{\pi }}\right)$$

By direct substitution, one can verify that Eq. (14) is indeed the solution of Eq. (6).

It is worth noting here that the series in Eq. (14) diverge as $\tau =1$. However, it can be used for calculations if $\tau <1$ (see details below).

The rest of this section is devoted to establishing, an important inequality, which allows one to estimate the pressure value.

Using the fact that^9,13^ $\left|{\mathfrak{D}}^{\nu }f(\tau )\right|\ll \sup|f|\frac{{\tau }^{-\nu }}{\Gamma (1-\nu )}$, it follows that, for an increasing function $p(\tau )$,

15 $${\mathfrak{D}}^{-1/2}p\ll 2p\sqrt{\frac{\tau }{\pi }}$$

Substituting the latter expression into Eq. (6) yields

16 $$p\ge \frac{1+2\lambda \sqrt{\tau /\pi }}{f(\tau )+2\lambda \sqrt{\tau /\pi }}$$

From this, it follows that, for $\tau = 1$,

17 $$p\left( 1 \right) \ge 1 + \frac{\sqrt \pi }{{2\lambda }}.$$

## Some examples

This section discusses three examples of the law of volume variation $f\left( \tau \right)$ that have important practical applications.

First, consider

18 $$f\left( \tau \right) = 1 - \sqrt \tau .$$

From Eq. (9),

19 $$\begin{aligned} a_{0} & = 1;a_{1} = 1;a_{2} = a_{1} \left[ {1 - \lambda \Gamma \left( {3/2} \right)/\Gamma \left( 2 \right)} \right];\cdots \\ a_{n + 1} & = a_{n} \left[ {1 - \lambda \Gamma \left( {\frac{n + 2}{2}} \right)/\Gamma \left( {\frac{n + 3}{2}} \right)} \right] \\ \end{aligned}$$

The solution in the series form (8) converges for an arbitrary value of $\lambda$ for all $0\le \tau \le 1$. This proves that the pressure value remains bounded for the moment of “collapse” of the gas volume.

Moreover, if $\lambda =\Gamma \left(\frac{n+3}{2}\right)/\Gamma \left(\frac{n+2}{2}\right)$, the series given by Eq. (8) ends on the *n*-th term and, hence, the solution can be written in a finite form. For instance,

20 $$\begin{array}{*{20}l} {\lambda = \Gamma \left( 2 \right)/\Gamma \left( {3/2} \right) = 2/\sqrt \pi ,} & {\quad p\left( \tau \right) = 1 + \sqrt \tau } \\ {\lambda = \Gamma \left( {5/2} \right)/\Gamma \left( 2 \right) = 3\sqrt \pi /4,} & {\quad p\left( \tau \right) = 1 + \sqrt \tau + \left( {1 - \frac{\sqrt \pi }{2}} \right)\tau } \\ \end{array}$$

Now consider the case of

21 $$f(\tau )=\left(1-{\tau }^{n+1/2}\right)/\left[1+\frac{\Gamma (n+3/2)}{\Gamma (n+1)}\frac{{\tau }^{n}}{\lambda }\right]$$

In this case, Eq. (8) renders a finite expression, namely:

22 $$p(\tau )=1+\frac{\Gamma (n+3/2)}{\Gamma (n+1)}\frac{{\tau }^{n}}{\lambda }$$

As can be seen, the pressure value remains bounded for the moment of “collapse” of the gas volume, too.

To conclude this section, consider the case, which is very important for practical applications, namely—the gas volume changes linearly—that is,

23 $$f\left( \tau \right) = 1 - \tau .$$

Two values of $\lambda$ are considered, namely: $\lambda =0.1$ and $\lambda =0.01$. This corresponds to the limiting cases with the characteristic parameters as follows: $\theta = 2 \times 10^{5} \ldots 2 \times 10^{6} \,{\mathrm{s}}$; $V_{0} = 1\,{\mathrm{m}}^{3}$; $\kappa = \left( {0.1\ldots 0.3} \right) \times 10^{ - 5} \,{\mathrm{kg}}/\left( {{\mathrm{m}}^{3} \cdot {\mathrm{Pa}}} \right)$; $F = 7\,{\mathrm{m}}^{2}$; $P_{0} = 1.5 \times 10^{5} \,{\mathrm{Pa}}$; $M = 29\,{\mathrm{kg/mol}}$; $R = 8310\,{\mathrm{J}}/\left( {{\mathrm{kmol}} \cdot {\mathrm{K}}} \right)$; $D = 10^{ - 9} \,{\mathrm{m}}^{2} /{\mathrm{s}}$; $T = 293\,{\mathrm{K}}$.

For the chosen values of $\lambda$, it is convenient to conduct pressure calculations using Eq. (14). The latter yields

24 $$p=\frac{1}{1-\tau }-\frac{2\lambda }{\sqrt{\pi }}\left[\frac{1}{(1-\tau {)}^{3/2}}\arctan\sqrt{\frac{\tau }{1-\tau }}-\frac{\sqrt{\tau }}{1-\tau }\right]+O\left[\frac{{\lambda }^{2}}{(1-\tau {)}^{2}}\right]$$

As follows from Eq. (24), the relative computational error is defined by the ratio of the third term of the series to the first one and is of the order of ${\lambda }^{2}/(1-\tau )$. Hence, the latter equation is not applicable for $\tau \to 1$.

From comparing the law of the gas volume change given by Eq. (23) with the laws given by Eqs. (18) and (21), it follows that, for any finite value of $\lambda$, it is possible to choose such a value of $n$ in Eq. (21) that all values of the function given by Eq. (23) will be between the values of the functions given by Eqs. (18) and (21). Hence, it becomes obvious that the pressure value remains bounded for the moment of “collapse” of the gas volume for the linear volume change as well.

Table 1 shows the computational results for the pressure value as a function of time obtained from using Eq. (24) with the relative computational error of $\sim 1$%. The results are compared with the estimate of the pressure value obtained by from the inequality (16).

**Table 1:** Dependence of the dimensionless pressure $p$ versus dimensionless time $\tau$ in the case of the linear change of the gas volume.

Eq	$\lambda$	$\tau$								
		0	0.2	0.4	0.6	0.8	0.9	0.95	0.98	1
24	0.1	1	1.24	1.62	2.32	4.11	8.63	–	–	–
16	0.1	1	1.23	1.60	2.23	3.60	5.94	6.94	8.44	9.86
24	0.01	1	1.25	1.66	2.48	4.91	9.66	18.86	44.87	–
16	0.01	1	1.25	1.70	2.47	4.81	9.13	16.57	31.02	89.62

As can be seen, inequality (16) renders the values close to the exact value (relative error < 10%) up to $\tau =0.7(\lambda =0.1)$ and $\tau =0.9(\lambda =0.01)$. In addition, this inequality renders the lower estimate of the pressure value for all values of $\tau$. One can draw a similar conclusion upon comparing (16) with the exact solutions for the first two cases considered in the beginning of this section. Hence, formula (16) can be recommended for practical applications.

To conclude this section, it is worth noting that, if the pressure is not a monotonously increasing function of time, the inequality sign in (16) may change to the opposite one.

## General discussion

It has been demonstrated so far that the solution for the pressure evolution at a gas–liquid interface is in the form of series with respect to fractional differ-integrals (derivatives of fractional order).

As has been already mentioned, problems similar to those treated here were considered earlier in connection with the study of the dynamics of growth and dissolution of gas bubbles taking into account many factors (e.g., surface tension, viscosity, etc.)^10–12^. Unfortunately, the said methods are quite laborious and able to render but approximate solutions. On the contrary, the method employed here not only renders exact solutions but is much less laborious. Moreover, a simple inequality for estimating pressure values has been established. The latter can be used by practicing engineers as a quick easy-to-use tool.

To conclude, it is worth noting here that the solution method considered in this paper, in addition to direct practical interest, seems to be useful from a methodological point of view, since the proposed approach is applicable to the study of more complex problems of the dynamics of phase interaction. For instance, the results, presented here, can be used to develop advanced non-equilibrium models of depressurisation in two-phase fluids.

## List of symbols

$a_{n} ,b_{n}$: Coefficients in fractional series
$$C,{\mathrm{kg}}/{\mathrm{m}}^{{3}}$$: Mass concentration
$c$: Dimensionless mass concentration
$$D,{\mathrm{m}}^{{2}} /{\mathrm{s}}$$: Diffusion constant
$F,{\mathrm{ m}}^{{2}}$: Gas contact area
$f$: Volume change function
$$k,n$$: Summation indices
$$M,{\mathrm{ kg}}/{\mathrm{mol}}$$: Molar mass
$$P,{\mathrm{ Pa}}$$: Pressure
$$p$$: Dimensionless pressure
$R,{\mathrm{J}}/\left( {{\mathrm{kmol}} \cdot {\mathrm{K}}} \right)$: Universal gas constant
$$T,{\mathrm{ K}}$$: Temperature
$$t,{\mathrm{ s}}$$: Time
$$V,{\mathrm{ m}}^{{3}}$$: Gas volume
$$x,{\mathrm{ m}}$$: Spatial variable

## Greek

$\Gamma$: The gamma function
$$\theta$$: Dimensionless temperature
$$\zeta$$: Dummy variable
$$\kappa ,{\mathrm{kg}}/({\mathrm{m}}^{3} \cdot {\mathrm{Pa}})$$: Henry coefficient
$$\lambda ,\xi$$: Dimensionless variables
$\nu$: Order of fractional differ-integration
$$\tau$$: Dimensionless time

## Special symbols

$\partial^{\nu }$: Fractional differential operator of order *ν*
$${\mathfrak{D}}$$: Fractional operator
